# TGR5 regulates portal perfusion pressure of the liver

**DOI:** 10.17179/excli2019-2077

**Published:** 2019-12-20

**Authors:** Ahmed Ghallab

**Affiliations:** 1 Forensic Medicine and Toxicology Department, Faculty of Veterinary Medicine, South Valley University, Qena, Egypt

## ⁯⁯⁯⁯⁯⁯⁯

***Dear Editor,***

Recently, Klindt and colleagues from the University of Düsseldorf published a study on the role of the bile acid receptor TGR5 in modulating portal pressure (Klindt et al., 2019[14]). TGR5 is activated by bile acids, leads to an intracellular increase of cAMP (Kawamata et al., 2003[10]; Keitel et al., 2019[13]; Maruyama et al., 2002[15]) and mediates cytoprotective effects (Keitel et al., 2009[11], 2015[12]; Merlen et al., 2020[16]; Perino and Schoonjans, 2015[18]; Perino et al., 2014[17]; Guo et al., 2016[5]). In their present study, the authors present a concept according to which activation of TGR5 in sinusoidal endothelial cells of the liver blocks the release of endothelin-1. Endothelin-1 is known to cause contraction of hepatic stellate cells via the ET_A_ receptor. Therefore, endothelin-1 leads to a narrowing of the liver sinusoids and an increase of portal pressure (Klindt et al., 2019[14]). Moreover, activation of TGR5 in hepatic stellate cells leads to internalization of the ET_A_R that also reduces the responsiveness to the contractile effect of endothelin-1. 

The prevalence of liver diseases currently increases (Jansen et al., 2017[9]; Ekhlasi et al., 2017[1]; Hudert et al., 2019[8]) and a better understanding of the responsible mechanisms is urgently needed to identify better strategies for therapeutic intervention (Svinka et al., 2017[20]; Godoy et al., 2016[3]; Ghallab et al., 2016[2]; Gogiashvili et al., 2017[4]). A particular challenge is that different cell types in the liver communicate in a complex manner, which leads to a situation, where the result of interventions is difficult to predict (Hoehme et al., 2010[7]; Hammad et al., 2014[6]; Schenk et al., 2017[19]). The present study of Klindt and colleagues represents an important milestone in understanding the pathophysiology of increased portal pressure. 

## Conflict of interest

The author declares no conflict of interest.
